# Supplementary material for: Vine-Winery Byproducts as Precious Resource of Natural Antimicrobials: In Vitro Antibacterial and Antibiofilm Activity of Grape Pomace Extracts against Foodborne Pathogens
Source: Microorganisms. 2024 Feb 21;12(3):437. doi: 10.3390/microorganisms12030437 (PMC10972293; doi:10.3390/microorganisms12030437)
Supplement: Supplementary file 1 [file microorganisms-12-00437-s001.zip › microorganisms-2846419-supplementary.pdf]

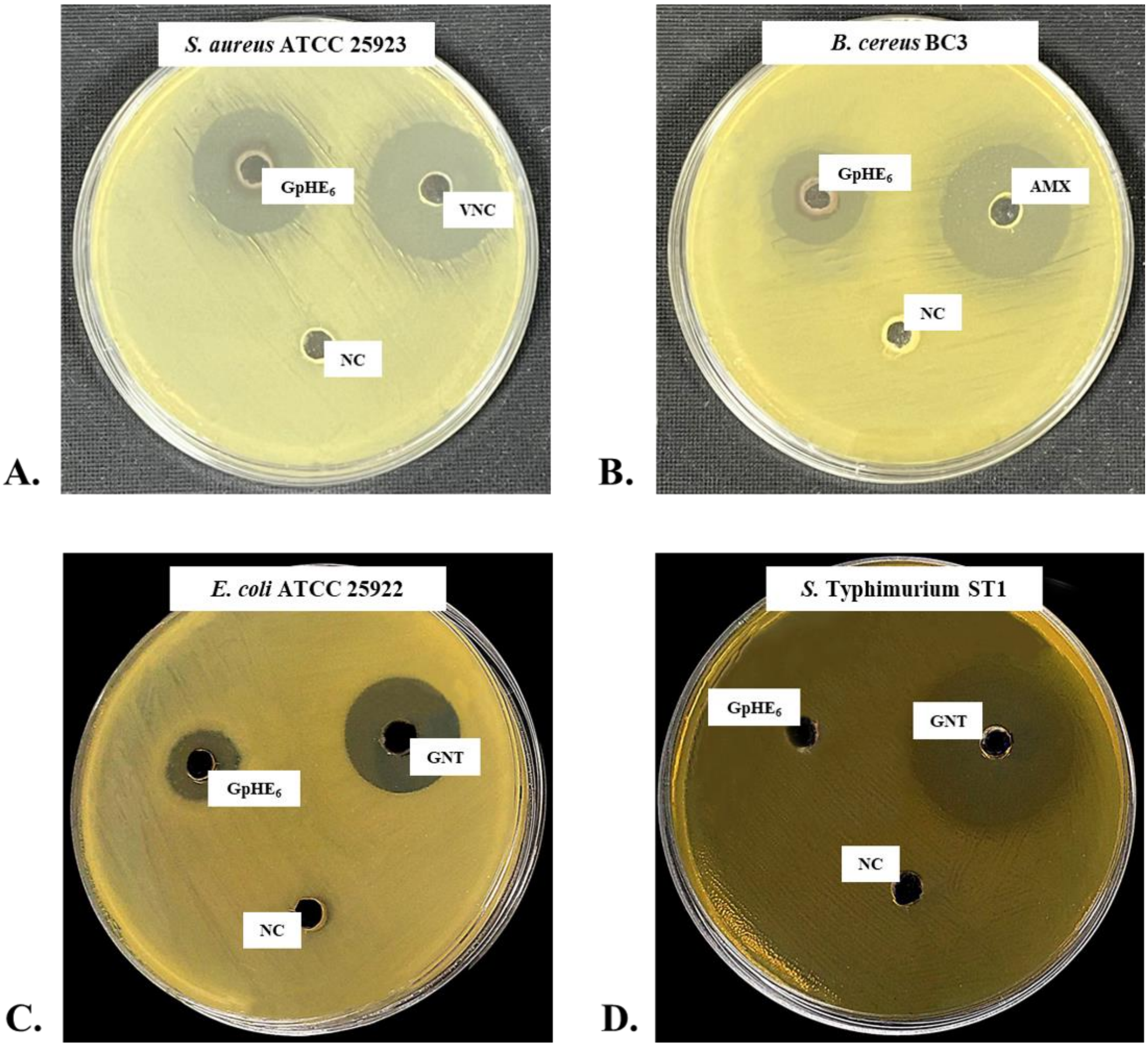

Figure S1. Images of *in vitro* antibacterial activity of hydro-ethanolic grape pomace extract against *Staphylococcus aureus* ATCC 25923 (A), *Bacillus cereus* BC3 (B), *Escherichia coli* ATCC 25922 (C) *Salmonella enterica* subsp. *enterica* serovar Typhimurium ST1 (D) foodborne bacteria. $GpHE_6$, grape pomace hydro-ethanolic extract n°6 (20 mg/well); VNC, vancomycin (0.5 mg/well); AMX, amoxicillin (5 mg/well); GNT, gentamicin (6 mg/well); NC, negative control (hydroalcoholic extraction buffer, composed of ethanol and distilled water at 50% v/v).

Table S1. Total polyphenols content of aqueous and hydro-ethanolic extracts of Aglianico (*V. vinifera* L.) grape pomace obtained by ultrasonic-assisted extraction.

N°	Extract	TPC (mg GAE g^{-1})
1	$GpAE_1$	7.9 ± 0.0 [a]
2	$GpAE_2$	12.2 ± 0.2 [b]
3	$GpAE_3$	9.3 ± 0.2 [a]
4	$GpAE_4$	15.7 ± 0.0 [b]
5	$GpAE_5$	8.7 ± 0.1 [a]
6	$GpAE_6$	16.5 ± 0.2 [b]
7	$GpAE_7$	12.3 ± 0.0 [a]
8	$GpAE_8$	28.5 ± 1.1 [c]
9	$GpHE_1$	24.4 ± 0.2 [c]
10	$GpHE_2$	51.9 ± 2.9 [d]
11	$GpHE_3$	26.5 ± 0.5 [c]
12	$GpHE_4$	58.2 ± 2.7 [e]
13	$GpHE_5$	26.6 ± 0.0 [c]
14	$GpHE_6$	57.1 ± 2.1 [e]
15	$GpHE_7$	26.9 ± 0.4 [c]
16	$GpHE_8$	50.4 ± 3.7 [d]

[1] TPC, total polyphenols content; GAE, gallic acid equivalents; GpAE, grape pomace aqueous extract; GpHE, grape pomace hydro-ethanolic extract.

[2] The results estimated by Folin-Ciocalteu assay are expressed in mg gallic acid (GAE) equivalents per g of dry solid matrix. Results were reported as mean values ± standard deviation.

[3] One-way ANOVA test was performed to evaluate statistical significance. Tukey's post hoc test ($p < 0.05$) allowed to examine the statistical significance for multiple comparisons. Different letters (a-e) indicate significant differences extracts; extracts with no significant differences receive the same letter.

Table S2. *In vitro* antibacterial activity of hydro-ethanolic grape pomace extract against *Staphylococcus aureus* ATCC 25923, *Bacillus cereus* BC3, *Escherichia coli* ATCC 25922 and *Salmonella enterica* subsp. *enterica* serovar Typhimurium ST1 foodborne bacteria.

Antibacterial agents	MDIZ (mm)			
	S. aureus ATCC 25923	*B. cereus* BC3	*E. coli* ATCC 25922	S. Typhimurium ST1
$GpHE_6$ (5 mg/well)	16.7 ± 1.7 ^{b*}	8.7 ± 0.5 ^{b****}	00.0 ± 0.0 ^{b****}	00.0 ± 0.0 ^{b****}
$GpHE_6$ (10 mg/well)	19.2 ± 0.9 ^{a}	9.3 ± 0.2 ^{b****}	7.2 ± 0.2 ^{c****}	00.0 ± 0.0 ^{b****}
$GpHE_6$ (20 mg/well)	21.2 ± 0.9 ^{a}	14.0 ± 0.8 ^{c****}	7.6 ± 0.4 ^{c****}	00.0 ± 0.0 ^{b****}
VNC (0.5 mg/well)	21.7 ± 2.9 ^{a}	-	-	-
AMX (5 mg/well)	-	32.7 ± 2.1 ^{a}	-	-
GNT (6 mg/well)	-	-	29.0 ± 0.8 ^{a}	18.5 ± 0.4 ^{a}

[1] MDIZ, mean diameter of the inhibition zone; $GpHE_6$, grape pomace hydro-ethanolic extract n°6; VNC, vancomycin; AMX, amoxicillin; GNT, gentamicin.

[2] Results were obtained by agar well diffusion method; triplicate assays with independent cultures. The mean diameters of inhibition zone are reported as mean values ± standard deviation (expressed in mm)

[3] One-way ANOVA test was performed to evaluate statistical significance. Comparison with positive control was analyzed by Dunnett's post hoc test ($p < 0.05$), using asterisks to indicate statistical significance respect to the positive control (**** $p < 0.0001$; * $p < 0.05$). Tukey's post hoc test ($p < 0.05$) allowed to examine the statistical significance for multiple comparisons between several tested volumes of extract $GpHE_6$ for each microorganism. Different letters (a, b) indicate significant differences between compared values; values with no significant differences receive the same letter.
